# Investigation of Tumor Suppressing Function of *CACNA2D3* in Esophageal Squamous Cell Carcinoma

**DOI:** 10.1371/journal.pone.0060027

**Published:** 2013-04-03

**Authors:** Yan Li, Cai-Lei Zhu, Chang-Jun Nie, Jiang-Chao Li, Ting-ting Zeng, Jie Zhou, Jinna Chen, Kai Chen, Li Fu, Haibo Liu, Yanru Qin, Xin-Yuan Guan

**Affiliations:** 1 State Key Laboratory of Oncology in Southern China, Sun Yat-sen University Cancer Center, Guangzhou, China; 2 Department of Clinical Oncology, The University of Hong Kong, Hong Kong, China; 3 Department of Clinical Oncology, the First Affiliated Hospital, Zhengzhou University, Zhengzhou, China; 4 Vascular Biology Research Institute, Guangdong Pharmaceutical University, Guangzhou, China; The Chinese University of Hong Kong, Hong Kong

## Abstract

**Background:**

Deletion of 3p is one of the most frequent genetic alterations in esophageal squamous cell carcinoma (ESCC), suggesting the existence of one or more tumor suppressor genes (TSGs) within these regions. In this study, one TSG, *CACNA2D3* at 3p21.1, was characterized.

**Methods:**

Expression of CACNA2D3 in ESCCs was tested by quantitative real-time PCR and tissue microarray. The mechanism of CACNA2D3 downregulation was investigated by methylation-specific polymerase chain reaction (MS-PCR). The tumor suppressive function of *CACNA2D3* was characterized by both *in vitro* and *in vivo* tumorigenic assays, cell migration and invasion assays.

**Results:**

*CACNA2D3* was frequently downregulated in ESCCs (24/48, 50%), which was significantly associated with promoter methylation and allele loss (*P*<0.05). Tissue microarray result showed that downregulation of CACNA2D3 was detected in (127/224, 56.7%) ESCCs, which was significantly associated with lymph node metastasis (*P* = 0.01), TNM staging (*P* = 0.003) and poor outcome of ESCC patients (*P*<0.05). Functional studies demonstrated that CACNA2D3 could inhibit tumorigenicity, cell motility and induce apoptosis. Mechanism study found that CACNA2D3 could arrest cell cycle at G1/S checkpoint by increasing expressions of p21 and p53 and decreasing expression of CDK2. In addition, *CACNA2D3* could upregulate intracellular free cytosolic Ca^2+^ and subsequently induce apoptosis.

**Conclusion:**

*CACNA2D3* is a novel TSG responsible to the 3p21 deletion event and plays a critical suppressing role in the development and progression of ESCC.

## Introduction

Esophageal squamous cell carcinoma (ESCC) is one of the most common cancers with a very poor outcome in China [1]. ESCC is characterized by its remarkable geographic distribution and high-risk areas include Northern China, Northern Iran and South Africa [2]. Although genetic alterations have been widely studied in ESCC, the precise mechanisms underlying esophageal carcinoma are poorly understood. Previous study in high-risk area suggested that genetic susceptibility might play a role in the pathogenesis of ESCC [3]. Like other solid tumors, the development of ESCC is also believed as a multi-stage process caused by the stepwise accumulation of genetic alterations. Comparative genomic hybridization and loss of heterozygosity studies found that deletion of 3p was one of the most frequent genetic alterations in ESCC [4]–[5], suggesting the existence of one or more tumor suppressor genes within these frequently deleted regions. Recently, single-nucleotide polymorphism (SNP)-mass array was applied to investigate the loss of heterozygosity at 3p in 100 primary ESCC cases, leading to the identification of four commonly deleted regions on 3p including 3p21 [6]. Two candidate TSGs, *PLCD1* at 3p22 and *PCAF* at 3p24 have been characterized for their tumor suppressing functions and mechanisms [7]–[8].

In the present study, another candidate TSG, *CACNA2D3* (calcium channel, voltage dependent, alpha-2/delta subunit 3) at 3p21.1, was characterized for its tumor suppressive function and mechanism. CACNA2D3 is an auxiliary member of the alpha-2/delta subunit family of the voltage-dependent calcium channel complex. Similar to CACNA2D2, it also regulates the influx of calcium ions entering the cell upon membrane polarization [9]. There are four calcium channel voltage-dependent alpha-2/delta subunit genes, *CACNA2D1* to *CACNA2D4* [10]. Frequent allele loss of *CACNA2D2* has been reported in lung, breast and other cancers [11]. One report indicated that *CACNA2D2* could mediate apoptosis in non-small cell lung cancer cells [12]. Another study found that promoter methylation of *CACNA2D3* was frequently detected in gastric cancer, which was associated with poor prognosis of the disease [13]. Growing evidence showed that Ca^2+^ signaling regulates diverse cellular processes such as fertilization, development, proliferation, learning and memory, and cell death [9]. Although *CACNA2D3* has been associated with the poor outcome of gastric cancer [13], the effect of *CACNA2D3* on ESCC development is not clear. In the present study, expression of *CACNA2D3* in ESCC was detected in primary ESCC and ESCC cell lines. Both *in vitro* and *in vivo* assays were used to characterize the potential tumor suppressive function of *CACNA2D3*.

## Materials and Methods

### Cell Lines and Primary Tumor Tissues

ESCC cell lines KYSE30, KYSE140, KYSE180, KYSE410, and KYSE510 were obtained from DSMZ, the German Resource Center for Biological Material [14]. Chinese ESCC cell line HKESC1 was kindly provided by Professor G Srivastava [15]. The cells were confirmed by cytogenetics as human origin in 2009 [16]. Patients with ESCC were selected consecutively from the surgical pathology archives of the Linzhou Cancer Hospital (Henan, China). None of the patients in the study had received preoperative radiation or chemotherapy. Tissue samples used in the study were approved by the Committees for Ethical Review of Research Involving Human Subjects in Zhengzhou University (Zhengzhou, China). Written informed consents for the original human work that produced the tissue samples were obtained.

### Tissue Microarray and Immunohistochemistry

Tissue microarray containing 300 pairs of primary ESCC (tumor and non-tumor tissues) cases was constructed as described in previous report [16]. Corresponding matched non-tumor tissues were obtained about 3 cm away from the tumor tissues (on average). None of the patients in this study had received follow-up radiation or chemotherapy. The age of patients ranged from 40–80 years at the time of surgery (median age: 59 years) and the male/female ratio was 1.3∶1. Immunohistochemistry (IHC) was performed using the standard streptavidin-biotin-peroxidase complex method. A 1∶100 diluted anti-CACNA2D3 (Novus Biologicals, Littleton, CO) antibody was used for CACNA2D3 detection. CACNA2D3 expression was compared between tumor and paired non-tumor tissues.

### Methylation Analysis

DNA extraction, bisulfate treatment, and MS-PCR was performed as described previously [7]. Briefly, FastStart Taq DNA Polymerase (Roche, IN) was utilized in the reaction, and the cycle number was 40 which was within the linear amplification range. The primers’ sequences of *CACNA2D3* for methylation analyses were: CAC-M-F: 5′-TATTTCGAAATTTAGGGTGTTTTTC-3′; CAC-M-R: 5′-GATACTA CCACCACGACTTAAACG-3′; CAC-U-F: 5′-GTGGTGTGTTTGGAGTAGTAGAT ATT-3′; CAC-U-R: 5′-CCAAACTTAAACACAATAAATCACA-3′.

### LOH Detection in Tissue Samples and Fluorescence in situ Hybridization (FISH)

Genomic DNA was extracted from tumor samples using TIANamp genomic DNA kit (TIANGEN, China). SNP site (rs589281) within *CACNA2D3* gene was PCR amplified and sequencing analyzed with primers (CAC-SNP-F1∶5′ TGTTGTGAT GATTAGGTGAG-3′; CAC-SNP-R1∶5′ CTGTGGAGAATCACCTAATTC-3′). The BAC probe was labeled and FISH was performed as previously described [17].

### Establishment of Cell Lines with Ectopic CACNA2D3 Expression

*CACNA2D3* was cloned into expression vector pcDNA3.1(+) and then transfected into KYSE30 and KYSE510 cells using lipofectamine™ 2000 (Invitrogen, Calsbad, CA). Empty vector was transfected into cell lines as negative controls. Stable colonies were screened by G418 at 500 µg/ml.

### RNA Extraction and Quantitative Real-time PCR (qRT-PCR)

RNA was extracted from tissues and cultured cells using Trizol (Invitrogen, Calsbad, CA). Reverse transcription was performed using SuperScript III (Invitrogen, Calsbad, CA). qRT-PCR was processed using SYBR Green Supermix and ABI7900HT Fast Real-Time PCR system (Applied Biosystems, Foster City, CA). (CAC-Fq: 5′-AGGGA TTCACGGTTATGCCTT-3′; CAC-Rq: 5′-GCCACACCTAAACCCTTTGTC-3′). Triplicate assays were done and values were normalized by the internal control (18S rRNA or *GAPDH*). PCR products were subjected to dissociation curve analysis to exclude amplification of nonspecific products. Quantitative of the PCR data was processed using the ΔC_T_ method as described previously [18]. 2-fold was considered the cutoff for ascertaining *CACNA2D3* downregulation.

### Antibodies and Reagents

Antibodies used: CACNA2D3 (Novus Biologicals, Littleton, CO), GAPDH, p53, p21, Cyclin E, Cyclin A, CDK2, E-cadherin, Caspase 3, and Caspase 8 (Cell Signaling Technology, Danvers, MA). siRNA targeting *CACNA2D3* was from Origene (MD).

### *In vitro* Tumor Suppressive Assays

The effect of *CACNA2D3* overexpression on cell proliferation was assessed by determining cell growth and viability with the use of CCK-8 (Dojindo, Japan). Foci formation assay and colony formation in soft agar was performed as described [16]. Triplicate independent assays were performed.

### *In vivo* Tumor Suppressive Assay

The study was approved by Institutional Animal Care and Use Committee of Cancer Cancer, Sun Yat-sen University. Animal experiments were performed in compliance with the guidelines for the Welfare of Experimental Animals in Cancer Center, Sun Yat-sen University. The tumorigenicity of cells was assayed by tumor formation in nude mice. *CACNA2D3*- and vector-transfected cells (2×10^6^) cells were subcutaneously injected into the flanks of 4-week-old male athymic BALB/c nu/nu mice (n = 5 for KYSE30 cells; n = 4 for KYSE510 cells), respectively. Tumor growth was checked twice a week. Following euthanasia, tumors were excised, fixed in 10% formalin and embedded in paraffin block for IHC study.

### Cell Migration and Invasion Assays

Transwell Permeable Support (24-well plate) (Corning Incorpotated, NY) was used to assess the rate of cell migration. Briefly, 4×10^4^ cells in 100 µl of serum-free medium were added to the upper chamber of the transwell insert. The lower chamber was filled with 600 µl medium with 10% fetal bovine serum. After 22 hr of incubation at 37°C, penetrated cells to the lower surface of the filter were fixed, stained with Crystal Violet, and counted under a microscope. The assay was repeated three times. For invasion assay, BioCoat™ Matrigel™ Invasion Chamber (24-well plate) (Becton Dickinson and company, Franklin Lakes, NJ) was used according to the manufacture’s protocol.

### Cell Cycle Analysis

Tested cells (1×10^6^) were fixed in 70% ethanol, stained with propidium iodide (Sigma-Aldrich, Germany) and DNA content was analyzed by Cytomics FC 500 (BECKMAN COULTER, Fullerton, CA). Cell cycle profile was then analyzed with Modfit LT 2.0. Three independent assays were performed.

### Measurement of Cytosolic Free Calcium

The intracellular free Ca^2+^ was measured by FACS with free-Ca^2+^-sensitive Fluo3-AM green fluorescence probe (Sigma-Aldrich, Germany). Cells were washed and incubated with 5 µM probe diluted in medium without serum for 60 min at 37°C. Cells were then washed and incubated with PBS for 30 min at 37°C in the dark to allow cellular esterases to cleave the acetoxymethyl group of Fluo 3-AM. Cells were trypsinized, washed and gently resuspended in PBS. Fluorescence intensity was measured by FACS analysis at an excitation wavelength of 488 nM and an emission wavelength of 530 nM. Three independent experiments were repeated.

### Apoptosis Assay

Cells were treated with Staurosporine (STS, Sigma-Aldrich, Germany) (0.25 µM for KYSE30, 0.1 µM for KYSE510), then collected and analyzed 15 hr later. Apoptosis was detected by Annexin-V-FLUOS Staining Kit (Roche, Germany) and *in situ* Cell Death Detection Kit (Roche, Germany) according to the manufacture’s protocol. Triplicate independent experiments were performed.

### Statistical Analysis

Statistical analyses were done using statistical software package (Version 16.0; SPSS, Inc., USA). Pearson Chi-square test was used to analyze the relationship between *CACNA2D3* expression and clinic-pathological features. Survival curves were generated according to the Kaplan-Meier method and statistical analysis was performed by Log-rank test. The Cox proportional hazards regression model was used to identify the independent prognostic factors. Students’ t-test was used to analyze data from function analysis, migration and invasion, TUNEL, and *in vivo* tumor formation. *P*<0.05 was considered statistically significant.

## Results

### *CACNA2D3 i*s Frequently Downregulated in ESCC

**Figure 1:**
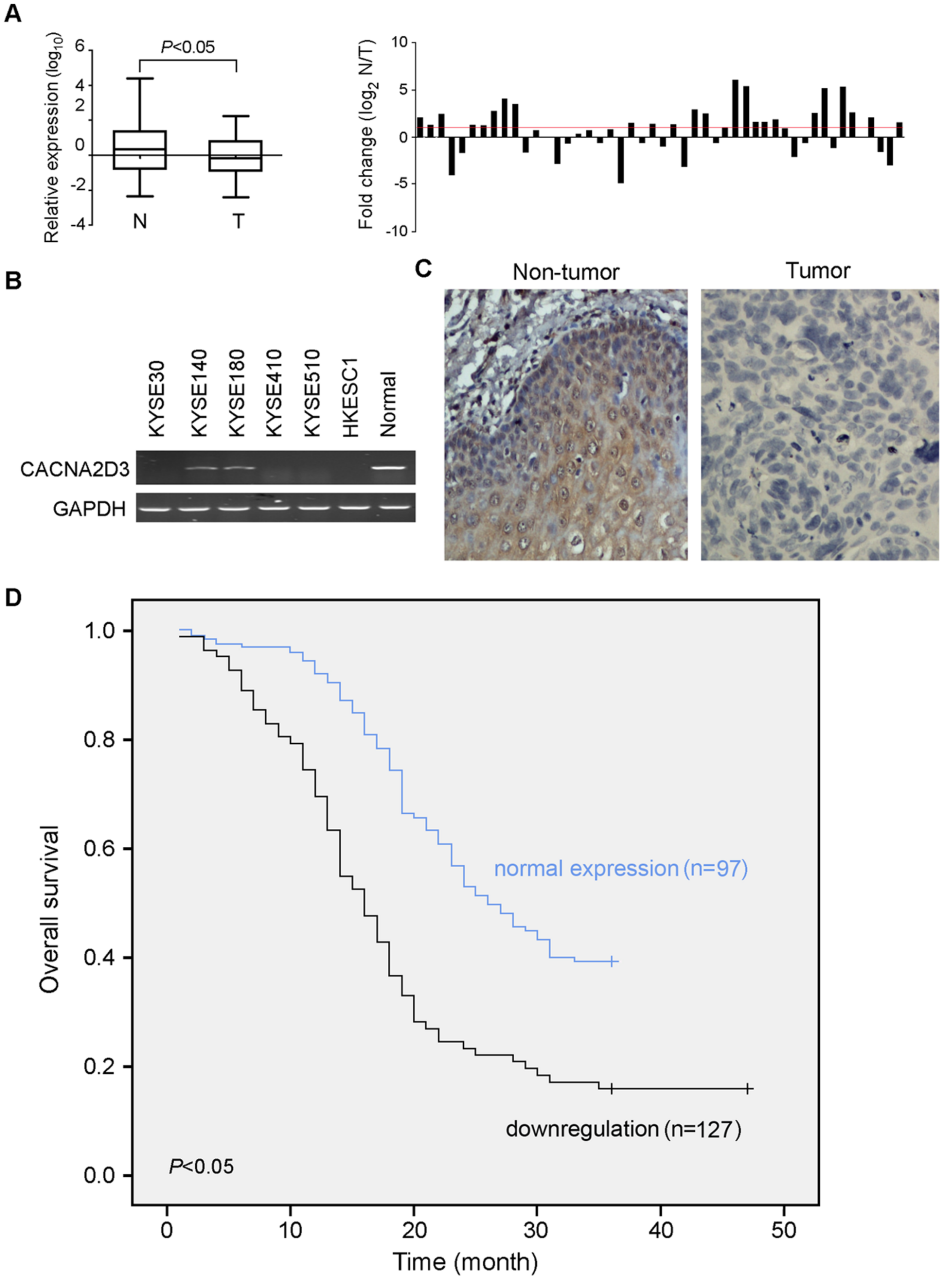
Downregulation of *CACNA2D3* in ESCC tumor tissues. (A) qRT-PCR was used to compare *CACNA2D3* expression between tumor (T) and non-tumor (N) tissues in 48 ESCCs. The result was normalized by *GAPDH*. Fold change (log2^N/T^) >1 (red line) was defined as “downregulation”. (B) Expression of *CACNA2D3* in ESCC cell lines was detected by RT-PCR, compared to normal esophageal mucosa. (C) Representatives of CACNA2D3 expression (brown color) detected by IHC in a pair of ESCC tumor tissue and paired non-tumorous esophageal tissue (20×objective). (D) Kaplan-Meier analysis shows that *CACNA2D3* downregulation is significantly associated with poor overall survival in 224 ESCC cases (*P*<0.05, Log-rank test).

Expression of *CACNA2D3* was compared between tumor and their paired non-tumor tissues in 48 ESCC patients by quantitative real-time PCR. The result showed that the downregulation of *CACNA2D3* was detected in 24/48 (50%) of ESCC tumor tissues compared with their paired non-tumor tissues (Fig. 1A). The average expression level of *CACNA2D3* was significantly reduced in tumor tissues compared with their paired non-tumor tissues (*P*<0.05). Downregulation of *CACNA2D3* was also detected in 4 ESCC cell lines KYSE30, KYSE510, KYSE410 and HKESC1 (Fig. 1B).

### Clinical Significance of *CACNA2D3* Downregulation in ESCC

Expression of CACNA2D3 in protein level was studied by IHC using a tissue microarray containing 300 pairs (tumor and non-tumor tissues) of primary ESCCs. Informative results were obtained from 224 pairs ESCCs. Noninformative samples included lost or samples with too few tumor cells. Downregulation of CACNA2D3 was detected in 127/224 (56.7%) informative ESCC tumor tissues compared with their paired non-tumor tissues (Fig. 1C). Higher expression of CACNA2D3 in tumor tissue was observed in 13/224 (5.8%) of ESCCs. Clinical association analysis demonstrated that downregulation of CACNA2D3 was positively correlated with lymph node metastasis (*P* = 0.01) and advanced clinical staging (*P* = 0.003, Table 1). Univariate survival analysis revealed that downregulation of CACNA2D3 was significantly correlated with poor overall survival (*P*<0.05, Table 2 and Fig. 1D). CACNA2D3 downregulation as well as other clinicopathologic features which were significant in univariate analysis (e.g. tumor differentiation, lymph nodes metastasis and TNM stage) were examined in multivariate analysis (Table 2). The result showed that the downregulation of CACNA2D3 was not an independent risk factor for overall patient survival (*P* = 0.054).

**Table 1:** Association between CACNA2D3 and clinicopathologic features of ESCC.

Clinicopathologic features	CACNA2D3 downregulation	*P*-value
***Age (years old)***		0.686
≤60	73/126 (57.9%)	
>60	54/98 (55.1%)	
***Gender***		0.786
Male	70/126 (55.6%)	
Female	57/98 (58.2%)	
***Tumor location***		0.273
Upper	23/48 (47.9%)	
Middle	90/149 (60.4%)	
Lower	14/27 (51.9%)	
***Differentiation***		0.068
Well	15/26 (57.7%)	
Moderate	93/147 (63.3%)	
Poor	19/51 (37.3%)	
***Tumor invasion***		0.409
T1+T2	13/27 (48.1%)	
T3+T4	114/197 (57.9%)	
***Lymph nodes metastasis***		**0.01**
N0	47/100 (47%)	
N1	80/124 (64.5%)	
***TNM stage***		**0.003**
I+IIa	34/79 (43.0%)	
IIb+III+IV	93/145 (64.1%)	

**Table 2:** Univariate and multivariate analysis of different prognostic variables in patients with ESCC.

Variables	Univariable analysis*		Multivariable analysis*	
	HR (95% CI)	*P*	HR (95% CI)	*P*
**Age**	1.193 (0.915–1.554)	0.192		
**Gender**	1.132 (0.867–1.477)	0.363		
**Tumor location**	0.916 (0.730–1.151)	0.452		
**Tumor invasion**				
T1+T2	1.00			
T3+T4	1.395 (0.937–2.076)	0.101		
**Differentiation**				
Poor	1.00			
Well or moderate	0.682 (0.503–0.924)	**0.014**	0.688(0.504–0.938)	**0.018**
**Lymph nodes metastasis**				
N0	1.00			
N1	1.868 (1.428–2.442)	**<0.001**	1.716(1.227–2.402)	**0.002**
**TNM stage**				
I+ II a	1.00			
II b+III+IV	1.158 (1.044–1.286)	**0.006**	1.063(0.854–1.324)	0.583
**CACNA2D3 downregulation**	1.393 (0.994–1.953)	**0.047**	1.442(1.026–2.027)	0.054

* Cox regression model; HR, Hazards ratio; CI, confidence interval.

### *CACNA2D3* Downregulation is Associated with Promoter Methylation and Allele Loss

**Figure 2:**
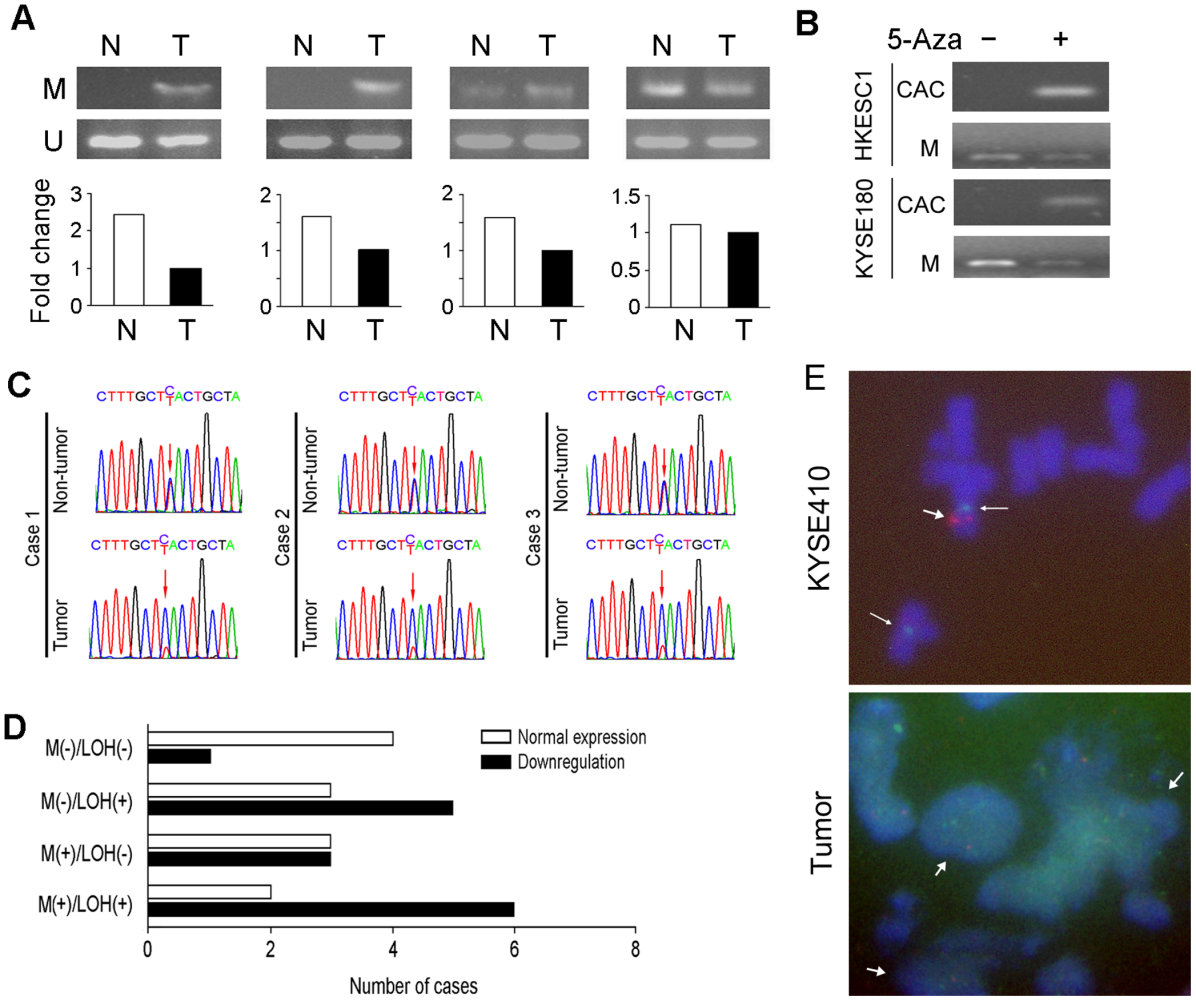
Downregulation of *CACNA2D3* is associated with promoter methylation and allele loss. (A) Representatives of *CACNA2D3* expression (*bottom*) and promoter methylation status (*upper*) in primary ESCC cases. (B) Restoration of *CACNA2D3* mRNA expression could be observed in HKESC1 and KYSE180 after demethylating agent 5-AZA treatment. The promoter methylation status was also compared. (C) Representative of allele loss at SNP site rs589281 in three ESCC tumors. The SNP site was indicated by red arrows. (D) Pattern of *CACNA2D3* expression, promoter methylation and allele loss in 27 informative ESCC cases. (E) 2 centrimere signals (green, indicated by narrow arrows) and 1 *CACNA2D3* signal (red, indicated by wide arrow) were detected in KYSE410 cells; Tumor cells with *CACNA2D3* deletion are indicated by arrows (100×objective).

As a putative mechanism for the downregulation of *CACNA2D3* in ESCCs, methylation status of a candidate CpG-rich promoter region was studied by methylation-specific PCR in 48 primary ESCCs. Promoter methylation was detected in 28/48 (58.3%) of tumor tissues (Fig. 2A). Downregulation of *CACNA2D3* was detected in 17/28 (60.7%) methylated cases, which is higher than that in cases without promoter methylation (7/20, 35.0%). In addition, expression of *CACNA2D3* could be restored in KYSE180 and HKESC1 cells after the treatment of the demethylation agent 5-AZA-dC (Fig. 2B). These data suggested that downregulation of CACNA2D3 was associated with hypermethylation in its promoter region.

In our previous study, LOH at the SNP site rs589281 within *CACNA2D3* gene was detected in about 50% of primary ESCC cases [6]. LOH at rs589281 site was also investigated in 48 ESCC samples and LOH was detected in 16/27 (59.3%) of informative cases with heterozygosity at the SNP site (Fig. 2C). Downregulation of *CACNA2D3* was detected in 11/16 (68.8%) LOH cases, which is higher than that in cases without allele loss (4/11, 36.4%). We next investigated the correlation of *CACNA2D3* downregulation with its allelic loss and hypermethylation in 27 informative cases. In 15 ESCCs with *CACNA2D3* downregulation, inactivation of CACNA2D3 was significantly associated with either methylation (n = 9) or LOH (n = 11), or both methylation and LOH (n = 6) (*P*<0.05, Fisher’s exact test, Fig. 2D). To validate qRT-PCR result, Fluorescence in situ hybridization (FISH) using BAC probe containing *CACNA2D3* was performed to check the deletion of 3p21 in 3 ESCC cell lines and 2 primary ESCC cases. The FISH result was consistent with qRT-PCR result. Loss of *CACNA2D3* allele could be observed in 2 cell lines (KYSE410 and KYSE510) and 1 primary ESCC tumor with the downregulation of CACNA2D3. (Fig. 2E).

### Tumor Suppressive Function of *CACNA2D3*

**Figure 3:**
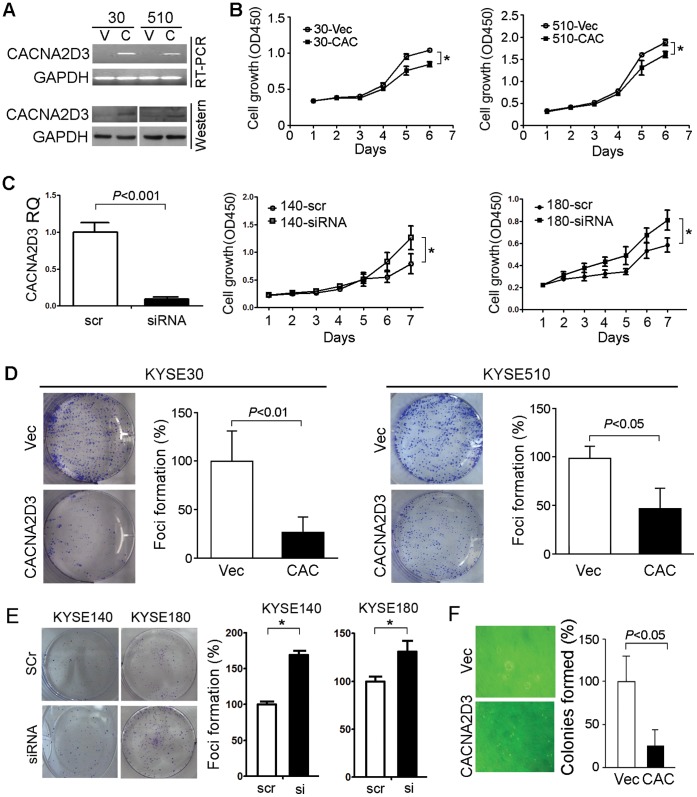
*CACNA2D3* inhibits tumorigenicity. (A) Detection of CACNA2D3 expression in *CACNA2D3*-transfected cells (30-*CAC* or 510-*CAC*) compared with vector-transfected cells (30-Vec or 510-Vec). *GAPDH* was used as loading control. (B) Cell growth rate was significantly inhibited in *CACNA2D3*-transfected cells compared with vector-transfected cells. (C) qRTPCR result of KYSE140 and cell growth rate was elevated in siRNA treated cells. (D, E) Ability of focus formation was decreased by *CACNA2D3* overexpression (D) and increased by silencing CACNA2D3 in cells (E). (F) Ability to form colony in soft agar decreased significantly in *CACNA2D3*-transfected cells compared with vector cells. *, *P*<0.05.

To characterize its tumor suppressive function, *CACNA2D3* was stably transfected into KYSE30 (30-*CAC*) and KYSE510 (510-*CAC*). Empty vector-transfected cells (30-Vec and 510-Vec) were used as controls. Expression of *CACNA2D3* was confirmed by RT-PCR and western blotting (Fig. 3A). Cell growth assay found that *CACNA2D3* could significantly inhibit cell growth in both tested cell lines (*P*<0.05, Fig. 3B). Similarly, *CACNA2D3* could significantly reduce focus formation (*P*<0.05, Fig. 3D) and colony formation in soft agar (*P*<0.05, Fig. 3F) in both tested cell lines, compared with vector-transfected cells. Silencing *CACNA2D3* could also reverse the results in KYSE140 and KYSE180 cells (Fig. 3C, 3E).

**Figure 4:**
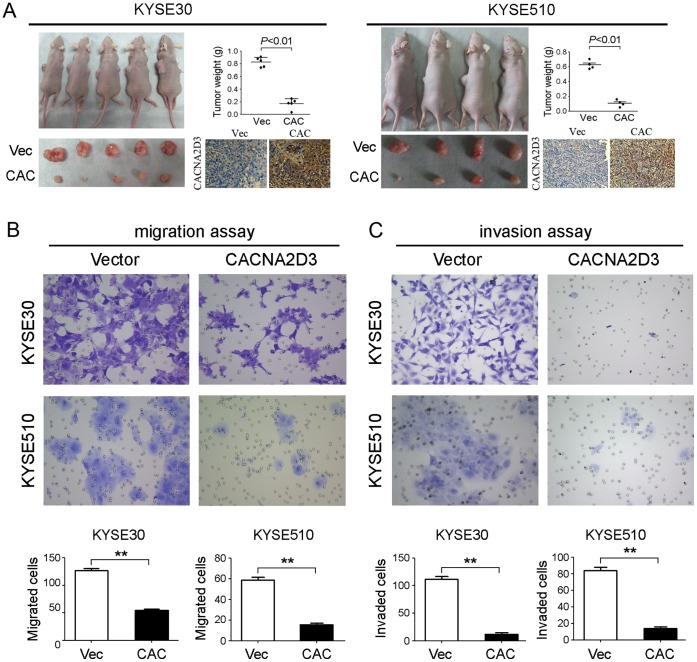
*CACNA2D3* inhibits tumorigenicity *in vivo* and cell motility *in vitro*. (A) Tumor formation in nude mice was inhibited by *CACNA2D3* in 30-*CAC* (left) and 510-*CAC* cells (right). Representatives of CACNA2D3 expression were detected by IHC in xenograft (20×objective). (B, C) Representative and summary of cell migration assay (B) and cell invasion assay (C) performed with *CACNA2D3* forced expression cells or vector cells. The results show that *CACNA2D3* inhibits cell migration and cell invasion (20×objective). **, *P*<0.001.

To further explore the *in vivo* tumor suppressive ability of *CACNA2D3*, tumor formation in nude mice was performed. Empty vector- and *CACNA2D3*-transfected cells were injected into the left and right flanks of nude mice, respectively. Twenty-four days after injection, mice were sacrificed and xenografts were excised for further analysis. The average weight of tumors induced by 30-*CAC* cells (0.168±0.080 g) was significantly decreased compared to the tumors induced by 30-Vec cells (0.825±0.072 g) (*P*<0.01, Fig. 4A). Similar result was also observed in KYSE510 cells (*P*<0.01, Fig. 4A). The IHC result showed that CACNA2D3 expression in *CACNA2D3*- transfected cells was much stronger than the vector controls (Fig. 4A).

### *CACNA2D3* Inhibits Cell Motility

Cell migration assay showed that 56.76% and 76.19% decrease in cells migrated through transwell were observed in the 30-*CAC* and 510-*CAC* cells, respectively, compared with control cells (Fig. 4B). Cell invasion assay found that cells invaded through Matrigel were also significantly decreased in 30-*CAC* (91.03%) and 510-*CAC* (75.07%) cells, compared with that in 30-Vec and 510-Vec cells, respectively (Fig. 4C).

### *CACNA2D3* Arrests Cell Cycle at G1/S Checkpoint

**Figure 5:**
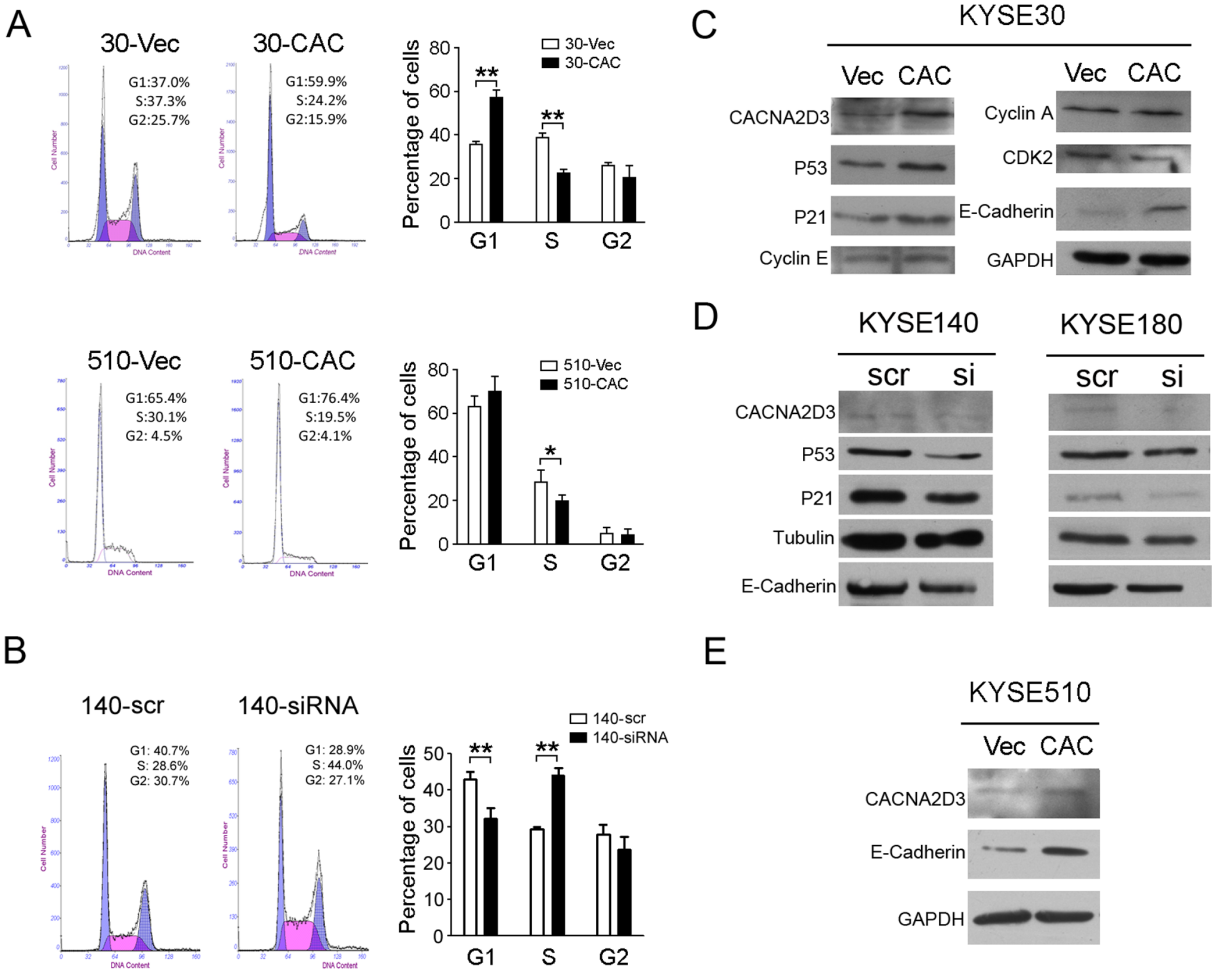
*CACNA2D3* arrests cell cycle at G1/S checkpoint. (A) Representatives and summary of DNA content of cells detected by flow cytometry. The results are expressed as mean±SD of three independent experiments. *, *P*<0.05, **, *P*<0.01. (B) Representatives and summary of DNA content in CACNA2D3 silenced cells. Scramble siRNA was used as a control. **, *P*<0.01. (C, D, E) Expression of proteins was detected in CACNA2D3 overexpressed cells (C, E) and silenced cells (D). GAPDH and tubulin were used as loading controls.

The cell cycle distribution between 30-*CAC* and 30-Vec cells was compared by flow cytometry. The result found that 30-*CAC* cells were arrested at G1/S checkpoint, manifested as an accumulation of cells in G1 phase (average 56.4%) and a decrease in S-phase cells (average 22.2%) compared to 30-Vec cells (G1 phase: average 36.9%; S-phase: average 37.2%) (Fig. 5A). Similar G1/S checkpoint arrest was also detected in 510-*CAC* cells (Fig. 5A). Silencing CACNA2D3 in KYSE140 cells caused a decrease in G1 phase cells (140-siRNA: average 32.1%) and increase in S phase cells (140-siRNA: average 44.1%) compared with 140-scr control (G1: average 42.9%; S: average 29.2%). (Fig. 5B). To investigate the potential mechanism of *CACNA2D3* in cell cycle arrest, expression of several key cell cycle regulators including p21, p53, CDK2, Cyclin A and Cyclin E were tested by western blotting. Increased expressions of p21 and p53, and decreased expression of CDK2 were detected in 30-*CAC* cells compared with 30-Vec cells (Fig. 5C). Silencing endogenous *CACNA2D3* with siRNA in KYSE140 and KYSE180 cells also decreased p21 and p53 (Fig. 5D). In addition, the expression of E-cadherin was increased in *CACNA2D3* overexpressed cells compared with vector cells (Fig. 5E), silencing *CACNA2D3* induced the decrease of E-cadherin expression (Fig. 5D).

### *CACNA2D3* Upregulates Intracellular Free Cytosolic Ca^2+^

**Figure 6:**
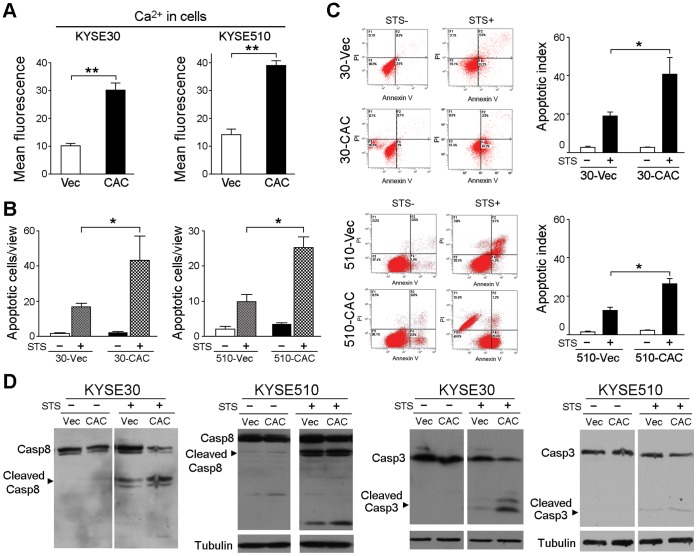
*CACNA2D3* induces apoptosis. (A) Intracellular Ca^2+^ level was compared between *CACNA2D3*-transfected and vector-transfected cells by FACS with Fluo 3-AM. A significant increase in fluorescence emission was observed in *CACNA2D3*-transfected cells compared with vector-transfected cells. **, *P*<0.001. (B) Summary of TUNEL assay performed with *CACNA2D3*-transfected cells or vector-transfected cells treated with or without STS. The results are expressed as mean±SD of three independent experiments. *, *P*<0.01. (C) Representative and summary of apoptotic index detected by flow cytometry in *CACNA2D3*-transfected cells or vector-transfected cells treated with or without STS. The results are expressed as mean±SD of three independent experiments. *, *P*<0.05. (D) Cleaved caspase-8 and caspase-3 were compared between *CACNA2D3*- and vector-transfected cells by western blot analysis. Tubulin was used as a loading control.

A previous study has reported that *CACNA2D2* could elevate intracellular Ca^2+^ level in non-small cell lung cancer cells [14]. In the present study, intracellular Ca^2+^ level in the KYSE30 cells after *CACNA2D3* transfection was measured by FACS. A significant increase in fluorescence emission was observed in 30-*CAC* cells compared with 30-Vec cells (*P*<0.01) (Fig. 6A), indicating that intracellular free cytosolic Ca^2+^ could be upregulated by *CACNA2D3*. Similar results were also observed in 510-*CAC* cells (Fig. 6A).

### *CACNA2D3* Induces Apoptosis

Since Ca^2+^ is able to mediate mitochondrial permeability transition and trigger apoptosis [19], FACS and TUNEL assays were used to compare apoptotic indexes between 30-*CAC* and 30-Vec cells. The TUNEL results showed that the number of apoptotic cells was similar between *CACNA2D3*-transfected and vector-transfected cells (Fig. 6B). However, the number of apoptotic cells was significantly increased in *CACNA2D3*-transfected cells (*P*<0.05) after STS treatment, compared to empty vector-transfected cells (Fig. 6B). Similar results were found by using FACS assay. After STS treatment, the apoptotic index, defined as the percentage of apoptotic cells (F2+F4), was significantly higher in *CACNA2D3* transfectants (*P*<0.05) compared with empty vector-transfected cells (Fig. 6C). Western blot analysis demonstrated that caspase-8 and caspase-3 were activated by the detection of cleaved forms of caspase-8 and caspase-3 (Fig. 6D).

## Discussion

In this study, we studied the downregulation of *CACNA2D3* and its tumor suppressive function and mechanism in ESCC. *CACNA2D3* is located at 3p21.1, a chromosomal region frequently deleted in lung [20], esophageal [6], [7], nasopharyngeal [21] and renal cell [22] cancers, suggesting that the existence of tumor suppressor gene(s) within the region that plays a critical role in the development and progression in various solid malignancies including ESCC. TSGs within 3p21 such as *RAR-β*, *RASSF1A* [23], *DLC1* [24] have been studied in ESCC. Here we described the characterization of another candidate TSG *CACNA2D3* at 3p21.

Downregulation of *CACNA2D3* was detected in 50% and 56.7% of primary ESCCs in mRNA and protein levels, respectively. Further study found that downregulation of *CACNA2D3* was significantly correlated with allele loss and promoter hypermethylation (*P*<0.05), indicating that DNA copy-number loss combined with promoter methylation played an important role in *CACNA2D3* downregulation. We noted that 2/8 of ESCC cases with both promoter methylation and allele loss retained expression of CACNA2D3 (Fig. 2D). We believe that this issue could be either caused by the heterogenicity of cancer or normal cell contamination (e.g. endothelial cells and lymphocytes). Actually, a small peak of lost allele could be observed in all tumor samples. We also noted that the promoter hypermethylation could be detected in 12/28 (42.8%) of methylation-positive ESCCs matched non-tumor tissues, which might be promoted by local environment. For example, Vasavi *et al.* found that patients with gastroesophageal reflux disease showed a high degree of hMLH1 hypermethylation, suggesting that local environment due to reflux might promote hypermethylation [25]. We believe “epigenetic filed defect” exists, which means slight methylation in the normal tissue but also a pre-cancerous change. Because intraepitherial neoplasia is frequently seen prior to ESCC, *CACNA2D3* could be a feasible biomarker to detect early change of the normal mucosa. Clinical significance study indicated that *CACNA2D3* could significantly inhibit lymph nodes metastasis (*P = *0.01) in ESCC. Kaplain-Meier analysis showed that overall survival rate of ESCC patients decreased as *CACNA2D3* was downregulated in tumor tissues.

To explore the tumor suppressive function of *CACNA2D3* in ESCC, functional analysis of *CACNA2D3* was performed by ectopic expression of *CACNA2D3* in ESCC cell lines KYSE30 and KYSE510. The results found that *CACNA2D3* could inhibit cell growth, focus formation, colony formation in soft agar and tumor formation in nude mice. Further study showed that *CACNA2D3* could arrest cell cycle at G1/S checkpoint by upregulating p53 and p21 expression and downregulating CDK2 expression. In addition, an elevated intracellular Ca^2+^ level was detected when *CACNA2D3* was introduced into cells, which was similar to the results of *CACNA2D3* in gastric cancer cells [13] and *CACNA2D2* in non-small cell lung cancer cells [12]. It has been reported that Ca^2+^ influx could promote activation of the transcription factor CREB (cAMP response element binding protein) leading to the cell cycle arrest in G1 phase via transactivation of p53/p21 signaling pathways [26]. Moreover, Ca^2+^ regulates the cell cycle through various signaling pathways including Ras [27], PTEN [28] and Rb [29] signaling pathways. It has been found that Ca^2+^ could mediate mitochondrial permeability transition and trigger apoptosis [19]. In this study, we found that *CACNA2D3* could significantly increase apoptotic index after STS treatment (*P*<0.05).

E-cadherin is the prototypic cadherin which is often lost partially or completely in epithelial tumors when they progress toward malignancy [30]. In this process, neoplastic cells have lost many of the epithelial characteristics and exhibit a highly invasive pattern [31]. In diffuse gastric cancer, E-cadherin is inactivated mainly through LOH, somatic mutations and promoter hypermethylation [32]. It has been reported that calcium signals regulate cell to cell adhesion through recruitment of cadherins and β-catenin into intracellular junction in fibroblasts [33]. Studies also found that reducing E-cadherin could induce disruption of the E-cadherin adhesion complex and correlated with elevated cell migration and invasion of different carcinoma cells [34]. In this study, we found that *CACNA2D3* could effectively inhibit cell motility in KYSE30 and KYSE510 cells, which might be associated with the upregulation of E-cadherin caused by the influx of Ca^2+^. In summary, our data indicate for the first time that downregulation of *CACNA2D3* is frequently detected in ESCC and associated with poor prognosis. Both promoter hypermethylation and allele loss contribute to the downregulation. We also demonstrate that CACNA2D3 has strong tumor suppressive function through the cell cycle arrest and induction of apoptosis.
